# Role of remote sensing, geographical information system (GIS) and bioinformatics in kala-azar epidemiology

**DOI:** 10.1016/S1674-8301(11)60050-X

**Published:** 2011-11

**Authors:** Gouri Sankar Bhunia, Manas Ranjan Dikhit, Shreekant Kesari, Ganesh Chandra Sahoo, Pradeep Das

**Affiliations:** Rajendra Memorial Research Institute of Medical Sciences (ICMR), Agamkuan, Patna, Bihar 800007, India.

**Keywords:** bioinformatics, geographical information system (GIS), rK39, support vector machine (SVM)

## Abstract

Visceral leishmaniasis or kala-azar is a potent parasitic infection causing death of thousands of people each year. Medicinal compounds currently available for the treatment of kala-azar have serious side effects and decreased efficacy owing to the emergence of resistant strains. The type of immune reaction is also to be considered in patients infected with *Leishmania donovani* (*L. donovani*). For complete eradication of this disease, a high level modern research is currently being applied both at the molecular level as well as at the field level. The computational approaches like remote sensing, geographical information system (GIS) and bioinformatics are the key resources for the detection and distribution of vectors, patterns, ecological and environmental factors and genomic and proteomic analysis. Novel approaches like GIS and bioinformatics have been more appropriately utilized in determining the cause of visearal leishmaniasis and in designing strategies for preventing the disease from spreading from one region to another.

## INTRODUCTION

**Fig. 1:**
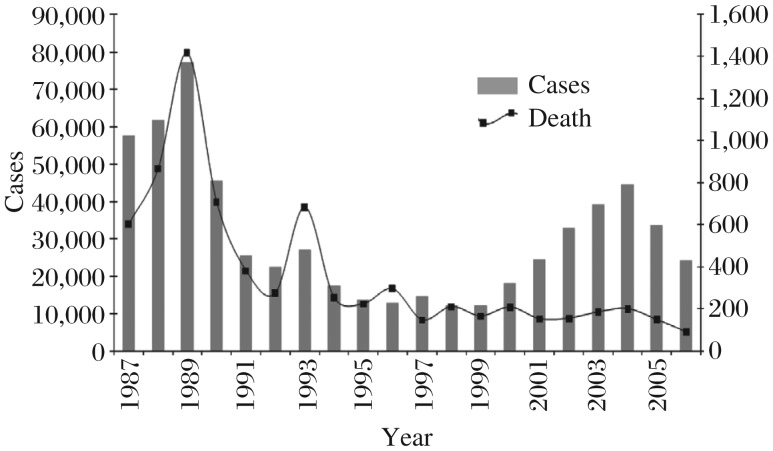
Time series plot of the estimated number of people with kala-azar in India (adopted from: Report on WHO, 2008)

On the Indian subcontinent, visceral leishmaniasis or kala-azar is a fatal vector-borne parasitic disease that has increased in incidence over the recent decades[1]-[2] and been considered an anthroponosis. *Leishmannia spp*. (*Phlebotomus argentipes*), the etiological agent of kala-azar, was first recognized in India in 1903[3]. Kala-azar epidemic in India started from Assam, spread to West Bengal and reached Bihar more than 100 y ago in the Purnea district[4]. India contributes more than 80% of the kala-azar cases in the South East Asian Region. According to the 2008 report (NVBDCP, 2008), kala-azar prevalence per 10, 000 population was estimated to be 3.43% in Bihar, 0.13% in West Bengal, 0.001% in Uttar Pradesh, and 1.36% in Jharkhand. A large epidemic of 100,000 cases of kala-azar occurred in Bihar in 1977[5]-[6] though the official figure was 18,589 only (unpublished data from the Department of Health, Government of Bihar, based on reports from the Primary Health Centers). The disease incidence has come down from 77,101 cases in 1992 to 24,209 cases in 2009 and deaths from 1,419 to 93, respectively. However, during 2010, the recorded cases were 3,344 with 2 deaths up to February, 2010. ***Fig. 1*** shows a time series plot of the number of people with kala-azar in India based on the data from the report by the World Health Organization (WHO), 2008. Visceral leishmaniasis has developed an epidemic cycle, taking place almost regularly every 15-20 years[7].

To achieve the elimination goal of the disease by year 2015, the Indian government is providing full supports apart from regular technical guidance. For example, timeliness and quality indoor residual spraying, complete treatment of patients as well as intensive social mobilization are being stressed upon. The significant factors in favor of making elimination goal are tools for early diagnosis, including rapid diagnostic test rK39, which can be used by trained peripheral health workers. However, the overall prevalence remains to be decreasing despite even relatively minor increases in kala-azar infection rate in the country. Consequently, proper strengthening of the information system between the district administration, Public Health Center (PHC) and village level is needed to bridge the gap between the outbreaks of the disease and failure to reach the information to the authority. In these cases, district authorities must be quicker to respond to patient needs, arrange better diagnostic tests and drugs, as well as implement good housing program.

More than a century ago, epidemiologist and health programmer were instigated to investigate the potential of maps for understanding the spatial dynamics of disease pattern and their association. Mapping can play an important role in both areas as it is an excellent means of communication. In order to be useful for resource planners, prediction of visceral leishmaniasis should include a spatial component. It is interesting to study and analyze the domain knowledge of remote sensing (RS), geographic information system (GIS) and bioinformatics and integrate them with the medical sciences to understand the advances and gaps.

RS and geographical bioinformatics system (GBS) are new fields of research and development that utilize expertise from two well-established fields, geoinformatics and bioinformatics. The term GBS refers to the application of computer science in the management of biological information using the spatial and temporal maps and databases to collect and study the complex patterns and analyze their effects. Researchers have agreed to use GIS applications to find and track large patterns, for example, geographic distribution of kala-azar and other diseases in human, animal and plant populations, etc. They are also exploring the potential of using RS and GIS to visualize and enhance the presentation of bioinformatics data, and to identify the opportunities of bioinformatics, and present them to the GIS research community. They are also exploring from another perspective the use of bioinformatics methodologies in GIS aiming at enhancing current GIS techniques, and identifying new approaches for pattern recognition and data analysis that could be used specifically for RS and GBS purposes.

The following is a short review of RS and GIS applications that analyze and visualize bioinformatics data to help bioinformatics experts study biological phenomena. These applications represent the solution for various bioinformatics databases from RS and GIS perspectives. The study is mainly focused on: 1) Exploring the future research in this new multidisciplinary field; 2) The “state of art” in the use of RS and GIS applied to endemics on visceral leishmaniasis (kala-azar); 3) Critically discussing the potential of RS and GIS for kala-azar epidemiology and epidemiologists, and some recommendations are presented.

## RS AND GLS APPLICATION ON BIOLOGLCAL PHENOMENA

### RS and visceral leishmaniasis or kala-azar

Sandfly, *P. argentipes* (vectors of Indian kala-azar) cannot be observed directly. For example, multispectral, microwave or thermal imagery is not used to observe sandfly directly from space, but can be used to identify the suitable environment or breeding places. The distribution pattern of sandfly and disease is highly affected by its physiography and ecology. The ecological and environmental factors may contribute to the transmission of the disease by enhancing the physiological activities of vectors and parasite. RS refers to the science of identification of earth surface features and estimation of their geo-biophysical properties using electromagnetic radiation as a medium of interaction. RS technologies, which allow the mapping of environmental variables, have been used in different epidemiological studies[8]-[12], but so far only rarely in the context of visceral leishmaniasis. Few studies are available that include the extraction of environmental indicators like meteorology, vegetation and altitude (***Table 1***). Neto *et al*.[13] developed an ecological niche model to delineate the distribution and potential risk zone of visceral leishmaniasis.

**Table 1:** Visceral leishmaniasis related to remote sensing

*Vector*	Location	Satellite/Sensor	References
*P. papatasi*	SW Asia	NOAA(AVHRR)	Cross *et al*., 1996[14]
*P. orientalis*	Sudan/ Africa	NOAA(AVHRR)	Thompson *et al*., 1999[57]
*L. chagasi, L. longipalpis*	NE Brazil	Landsat-5 (TM)	Thompson *et al*., 2002[57]
*P. orientalis*	Sudan/Africa	SPOT	Elnaiem *et al*., 2003[25]
*L. longipalpis*	Bahia/Brazil	Bio-climatic variable, SRTM	Neito *et al*., 2006[85]
*P. argentipes*	Bihar/India	IRS LISS-III	Sudhakar *et al*., 2006[17]
*L. chagasi*	Teresina/Brazil	Landsat-5 (TM)	Neto *et al*., 2009[13]
*P. argentipes*	India	SRTM, NOAA	Bhunia *et al*., 2010[8]
*P. argentipes*	NE India	NOAA(AVHRR)	Bhunia *et al*., 2010[89]
*P. alexandri*	Middle East	NOAA(AVHRR)	Colacicco-Mayhugh *et al*., 2010[86]
*L. longipalpis*	Brazil	LANDSAT (TM)	Werneck &Maguire, 2002[39]
*L. spp.*	Brazil	LANDSAT (TM)	Aparício & Dantas, 2003[87]

Nevertheless, RS data related to kala-azar studies have been focused on medium to low resolution satellite data, such as Landsat's Multispectral Scanner (MSS) and Thematic Mapper (TM), the National Oceanic and Atmospheric Administration (NOAA)'s Advanced Very High Resolution Radiometer (AVHRR), and France's Système Pour I'Observation de la Terre (SPOT). A list of different earth observing satellite sensors are provided to introduce readers to the types of the sensors that can be used to determine responsible environmental factors for visceral leishmaniasis (***Table 2***). Most information obtained by satellite is correlated to vegetation, utilizing Normalized Differential Vegetation Index (NDVI), an index obtained from operations with spectral bands. In kala-azar epidemiology, utility of high spatial resolution is rare; microwave imagery is almost absent. However, this technology has been widely used for health issue monitoring, disease re-emergence explanation, prediction and risk map amplification[9],[14] by applying a combination of weather data and AVHRR-GAC data to forecast the geographic and seasonal distribution of *Phlebotomus papatasi* in southwest Asia. A computer model was generated using the occurrence of *P. papatasi*, as a dependent variable and the mean synoptic weather data (114 meteorological stations) as independent variables. Mean monthly NDVI data from the NOAA-AVHRR were calculated for the period 1982-1994. The result of the frequency of NDVI levels versus the probability of vector occurrence was then used to establish NDVI limits for vector presence (0.00±0.06). This result has provided useful information on the spatial and temporal distribution of *P. papatasi* in the region.

**Table 2:** Earth observing satellite sensors used to determine responsible environmental factors for mapping visceral leishmaniasis or Kala-azar

Leishmania species	Vectors	Country: area	Environmental parameters	Satellite: Sensor	Technique Base map	References
*L.i. chagasi*	*Lu. longipalpis*	Brazil: Minas Gerais	Vegetation, Altitude, Hydrographic basin			Margonari *et al*., 2006[24]
*L. chagasi*	*Lu. longipalpis*	Brazil: Teresina	Vegetation	Landsat 5 TM	NDVI	Neto *et al*., 2009[13]
*L.i. chagasi*	*Lu. longipalpis*	Argentina: Misiones	Land cover characteristics	IKONOS	Supervised classification	Fernández *et al*., 2010[88]
*L. donovani*	*Ph. martini* and *Ph. orientalis*	East Africa	Vegetation Index and midday Land Surface Temperature	NOAA (AVHRR)	NDVI, LST	Gebre-Michael *et al*., 2004[18]
*L. donovani*	*Ph. argentipes*	India: Bihar	Vegetation index, land cover features	IRS-1C LISS III	NDVI, Supervised classification	Sudhakar *et al*., 2006[17]
*L. donovani*	*Ph. argentipes*	India: Bihar	Eco-enviromental parameters	NOAA (AVHRR)	Supervised classification	Bhunia *et al.*, 2010[89]
*L. donovani*	*Ph. argentipes*	India: Bihar	Vegetation index, altitude	SRTM, Landsat TM 5	DEM, NDVI	Bhunia *et al*., 2010[8]
*L.i. chagasi*	*Lu. longipalpis*	Brazil: Bahia: Sanitarion de Barra	Vegetation	NOAA (AVHRR)	NDVI	Bavia *et al*., 2005[20]
	*L braziliensis*	Argentina: Formosa: Las Lomitas	River, Vegetation	Landsat 5 TM Landsat 7 ETM+	Visual identification	Salomon *et al*., 2006[27]
*L. donovani*	*Ph. orientalis*	Sudan	Vegetation Index and Land Surface Temperature	NOAA (AVHRR)	NDVI, LST	Thomson *et al*., 1999[43]
*L. chagasi*	*L longipalpis*	Brazil: Ceara: Caninde	Vegetation Indices, Land cover characteristics	Landsat TM	NDVI, TC, Unsupervised classification (ISODATA)	Thompson *et al*., 2002[57]
*L. chagasi*	*Lu. longipalpis*	Brazil: Bahia	Altitude and Climate	WorldClim bioclimate variables, Global digital elevation model (GTOPO 30) program	Interpolation of recorded climate data from different weather stations	Nieto *et al*., 2006[85]
*L. major, L. donovani*	*P. Papatasi, Paraphlebo-tomous alexandri*	Middle East	Elevation, precipitation, land cover, and WorldClim bioclimatic	AVHRR (NOAA)	Ecological Niche Model	Colacicco-Mayhugh *et al*., 2010[86]
*L. infantum*	*Lu. migonei*	Argentina: La Banda, Santiago del Estero		Google Earth	Map visualization	Salomón *et al*., 2010[91]
*L. donovani*	*P. papatasi*	SW Asia	Vegetation, Weather data	NOAA(AVHRR)	Computer modeling using AVHRR-GAC data	Cross *et al*., 1996[14]
*L. donovani*	*Ph. orientalis*	Africa: Sudan: Gedaref State	Vegetation status, Wetness Index, Altitude	USGS data(hydrology, topography) SPOT	DEM, Slope, aspect, compound topographic index flow accumulation, NDVI	Elnaiem *et al*., 2003[25]

### RS studies in sandfly habitat mapping

Visceral leishmaniasis transmitted by sandflies [*Phlebotomus argentipes* (*P. argentipes*)] was reported as a health hazard for troops deployed in India[15]. The spatial distribution of sandflies is poorly understood, and knowledge of *P. argentipes* breeding sites remains scanty[16]. Comparison of *P. argentipes* densities between endemic and non-endemic to visceral leishmaniasis with different land use/land cover characteristics (LULC) derived from Indian Remote Sensing (IRS) Linear Imaging Self Scanning III (LISS III) data in North India showed that the endemic areas had a higher percentage of waterbody, succulent vegetation and high vector density compared to the non-endemic areas[17]. The distribution of *Ph. Martini* and *Ph. orientalis* using NDVI, midday land surface temperature, soil, and agro-ecological characteristics has been modeled[18]. This map was able to identify accurately all the areas where sandflies were present and useful for the health authorities in prioritizing their visits to specific sites. Using a predictive model of vector density in relation to NDVI, it was observed even at a null NDVI index. With increasing values of NDVI, the number of sandflies tended to decrease[19],[20] and, similarly, NDVI values have advocated the low and high probability of *P. papatasi* zone[14].

### GIS in kala-azar control programme

**Fig. 2:**
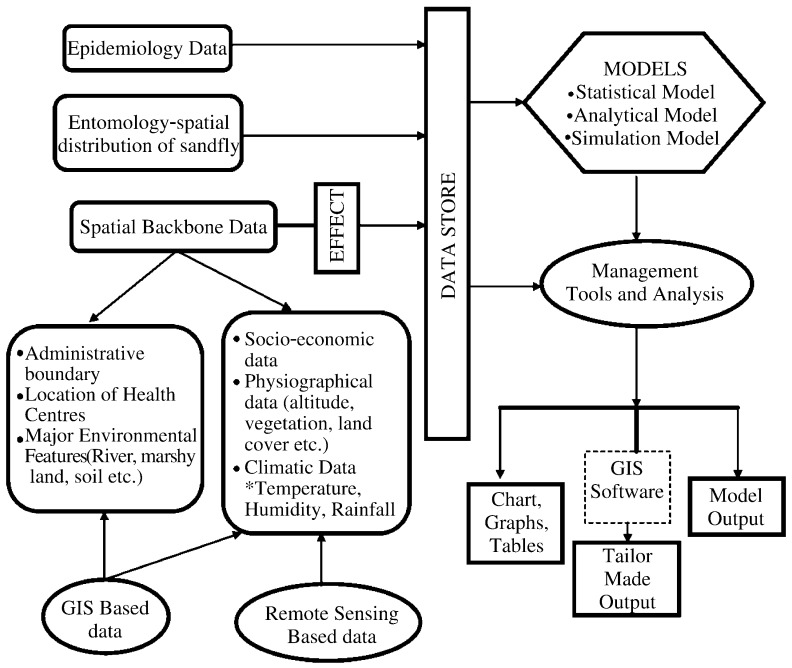
Decision support system of kala-azar disease analysis through remote sensing and GIS technique

The GIS is a special type of information systems and consists of hardware, software, data, people, and procedures that work together to produce quality information. It is the key tool to map the vectors and evaluate environmental factors that influence spatial and temporal distribution of vector/insects of several projects in public health and epidemiology[21]. One of the most useful functions of GIS in kala-azar epidemiology continues to be its utility in basic mapping[22]-[24]. While visual analyses (mapped evidence) and exploratory analyses are by and large adequate for epidemiologists, the formal testing of certain hypotheses or the estimation of relationships between measures of disease incidence and environmental covariates require quantitative modeling of disease distribution (***Fig. 2***).

GIS applications related to kala-azar have been introduced and used in the surveillance and monitoring of diseases[10], in environmental health[8],[17],[25], quantifying environmental hazards and their influence on public health[26]-[28], and for policy and planning purposes[29]-[31]. In India, GIS systems have been used in vector control research[32]-[34] for studying and mapping of non-communicable diseases[35]-[37]. Geographical epidemiological studies, in which health and environmental exposure data are analyzed in fine geographical detail, represent an important new approach[38].

The aims and purposes of disease mapping in the context of kala-azar are: 1) To describe the spatial variation in disease incidence for the formulation of etiological hypotheses; 2) To identify areas of unusually high risk in order to take preventive action; 3) To provide a reliable map of disease risk in a region to allow better resource allocation and risk assessment.

GIS may also involve more sophisticated spatial analysis of disease occurrence and contributing environmental factors. However, the spatial and geo-statistical analysis capability of GIS is argued to be rather limited and, therefore, the value of GIS for spatial epidemiology should be critically assessed and discussed. For example, the display of kala-azar cases vis-à-vis sandfly density of kala-azar vector, *P. argentipes*, and locations on a vegetation layer[8] may reveal the association between cases or sandfly and the distribution of vegetation covers[39] used spatial analysis of visceral leishmaniasis cases in northern Brazil to identify proximity to forests and pastures as the major risk factors.

### Research perspective: RS, GIS, and kala-azar

Over the past decade, the focus of some of this assistance has been in the provision of GIS hardware, software and training. In theory, GIS can be a very effective tool in combating visceral leishmaniasis or kala-azar; however, in practice there have been a host of challenges to its successful use.

#### Mapping visceral leishmaniasis (kala-azar) incidence/prevalence

This is the most basic application and involves mapping the incidence/prevalence of visceral leishmaniasis (kala-azar) over some geographic area[40]. The focus is on examining past trends as well as the present situation[41], and typically does not include any statistical analysis with the possible exception of correlating visceral leishmaniasis (kala-azar) incidence/prevalence with population in order to calculate populations at risk. The goal with these studies is to see if any obvious patterns exist or not.

#### Mapping of relationships between visceral leishmaniasis (kala-azar) incidence/prevalence and other potentially related variables

The timeframe is still on past trends and the present situation. The goal of these studies is to see if any relationships exist between visceral leishmaniasis (kala-azar) incidence/prevalence and a host of other variables including: temperature, rainfall, etc.[42]-[45]; land use/land cover; elevation; demographics (age and gender); population movement[8],[17],[46],[47]; climate change[48],[49]; breeding sites[10],[50] and control programs[10],[51],[52]. In most cases, these studies involve testing to see if any statistical relationships exist.

#### Using innovative methods of collecting data

The most important limitations of GIS are data gathering. The literature mostly deals with RS in the form of aerial photography and satellite imagery, but they have not mentioned the scale and the truthfulness of data[53],[54]. Currently, various GIS data are based on different scales. When those GIS data are overlaid with other GIS data in different scales, many problems will be encountered in the accuracy domain[55]. As noted earlier, spatial statistical analysis is a newly developing field and has no agreed upon or standard methodologies[56]-[58].

#### Modeling visceral leishmaniasis (kala-azar) risk

Modeling approaches have proven to be highly relevant in kala-azar studies that include either inductive/empirical models or deductive/theoretical models. There is a need for tailored capacity-building activities and attention to performance-based programs expands its response to the epidemic in the direction for a stronger multi-sector response. Risk models typically use many of the same variables discussed above-the difference being that statistical relationships are established between visceral leishmaniasis (kala-azar) incidence/prevalence (the dependant variable) and a range of independent variables in an effort to predict future cases of visceral leishmaniasis (kala-azar)[59] and develop multilevel modeling at different spatial scales to investigate disease transmission. Neto *et al*.[13] have developed two predictive ecological niche models within a geographic information system using genetic algorithm rule-set prediction (GARP) and the growing degree day (GDD)-water budget (WB) concept to predict the distribution and potential risk of visceral leishmaniasis in the State of Bahia, Brazil.

#### What RS and GIS software is being used?

The referred literature is probably not the best place to get an idea of the type of RS and GIS software that is used by those dealing with visceral leishmaniasis (kala-azar) research and control. This is because the software used by visceral leishmaniasis (kala-azar) researchers is typically different from that used by public health practitioners. This is the most important issue dealing with the problem that most image processing software [ERDAS Imagine, Multispec©, GRASS (www.grass.itc.it), MrSID GeoExpress View (www.lizardtech.co)] typically originates from the United States or Europe. In some cases, this results in problems getting copies of the software as well as getting support for the software. Some GIS software like ArcGIS® (ArcMap, ARC/INFO), Integraph GeoMedia® (www.integraph.com), HealthMapper and research analyst disseminate the use of GIS as tool for analysis and problem solving. The package offers simplified tools and interfaces to efficiently carry out bio-statistical and geographical analysis to support decision-making in kala-azar control program strategy. Such information when mapped together creates a powerful tool for monitoring and management of disease and other public health programs.

### Bioinformatics and GIS

Bioinformatics, RS and GIS are the upcoming fields not only in disease epidemiology but also in every field of life. The application of these technologies has been documented and applauded for their accuracy, rapidity and economic considerations. Bionformatics is the science that deals with the collection, organization and analysis of large amount of biological data using advanced information technology such as networking of computers, software and databases[60]. It expands simple searches of computer databases into new ways to merge data and divulge answers to complex queries in environmental studies, molecular biology, and climate change. Bioinformatics will alter epidemiological studies, as cataloguing of collections is completed and methods are developed to investigate associations among different datasets. Researchers in bioinformatics generally look at very small patterns, motif, genomic map and structure that might predispose an organism[61] whereas, GIS tools can be used by researchers to find and track large patterns, for example, geographic distribution of kala-azar[62], showing the relationship between location of disease and land cover, or soil types, in the future.

Bioinformatics and GIS have much in common, most notably large dedicated databases, visualization technique, pattern recognition, digital maps and analysis. Researchers are facing dreadful challenges in trying to recognize and analyze meaningful patterns in the rapidly growing volumes of data and information[63]. Both disciplines rely heavily on the use of maps (genomic and proteomic maps in case of bionformatics and geographical maps in case of GIS) for abstract representations of data. The utilization of bionformatics methodologies in geographical information system aims at enhancing current GIS techniques and identifying new approaches to recognize pattern and data analysis that could be used specifically for GBS purposes[64]. For dynamic representation, GIS methodologies can be applied for efficient biological database management while developing a database. The marine microbial diversity database provided GIS option with an interface for selecting a particular sampling location along with genome sequences and their details denote the fusion of GIS with bionformatics[65]. In GIS, provenance information includes depiction of the lineage of the data product including description of the data source, the transformations used to derive it, reference to the control information and mathematical transformations of the coordinates[66]. Lineage Information Program (LIP) follows a data-oriented provenance technique for GIS and is used for informational purposes, update stale data, regenerate and compare data[67],[68]. We consider that if this lineage information is stored and recorded in the machine readable as bionformatics, it can be applied for RS data dissemination and management to realize a functional model of kala-azar disease. Monitoring, quantifying, and predicting the human-health consequences related to kala-azar through environmental management of biological information uses the spatial and temporal maps, and databases to collect and study the complex patterns and analyze its effects.

In the past, GIS and spatial epidemiology focused on finding and monitoring large-scale, population-based occurrences such as disease clusters, outbreaks of infection, or possible associations between vector, pathogen and environmental factors. The amalgamation of genomics and proteomics with GIS and spatial epidemiology has the potential to provide an immense breakthrough. This will permit us to do a far better job of monitoring, quantifying, and predicting human-health consequences connected with the environment.

**Fig. 3:**
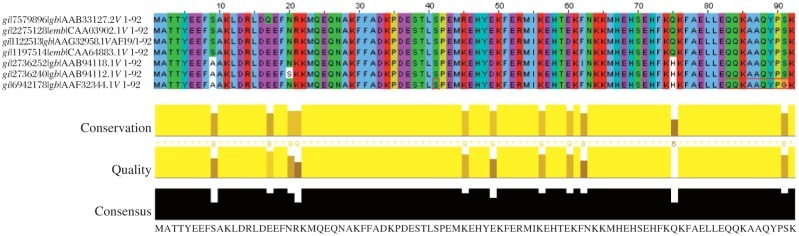
Multiple sequence alignments of KMP11 protein sequences from seven different *Leishmania* strains generated with the program of Clustal W program[92]. It represents conserved sequence regions colored by residue across a group of sequences hypothesized to be evolutionarily related. The interactive highlighting lower part of the image in yellow and black color shows the conservation, quality and consensus region corresponding to the amino acid or codon throughout the sequences.

Software has been developed as tools of bionformatics to analyze micro-level data like nucleotide or amino acid sequence data and extract biological information. Gene prediction software (http://www.scfbio-iitd.res.in/chemgenome/chemgenomenew.jsp and http://www.genomethreader.org/) and ‘Sequence alignment software’ (http://blast.ncbi.nlm.nih.gov/Blast.cgi & http://www.ebi.ac.uk/tools/clustalw2) are examples of some of the software developed for bionformatics. Multiple sequence alignment of KMP11 amino acid sequences from seven various *Leishmania* strains are shown in ***Fig. 3***. Gene prediction software is used to identify a gene with a long gene sequence. Efficient software like ArcGIS may become useful if it is utilized for visualizing, analyzing and querying the genome database better than the presently available techniques[69]. Thus, GIS function development technique can be replicated to make the available software more efficient. A general concept, methodology and tool was developed by Dolan *et al*.[69] for the display of geographic data to develop a Genome Spatial Information System (GenoSIS) for spatial display of genomes. Schweizer[70] showed the spatial aspects of bioinformatics through imaging and image processing tools that could be used for pattern recognition and analysis. FBK (http://mpba.fbk.eu/en/home) developed novel mathematical methods and ICT platform that may hook up the physiographical patterns of disease with high dimensional data, now available for functional genomics (e.g. DNA microarrays, SNPs, proteomics, deep sequencing) with spatially epidemic simulation system and geo-databases of environmental factors and socio-demographic data. Programs like GenoSIS and tools of FBK put forward the step in the integration of GIS and bionformatics, which can be used for bringing dynamism in the database search and predictive models on complex spatio-temporal patterns. Moreover, another aspect of genome research is molecular modeling and 3-D visualization. The maps developed by X-ray crystallography and nuclear magnetic resonance (NMR) spectroscopy techniques, which are preserved in the form of a Protein Data Bank (PDB), aid to solve protein structure determination. Similarly, in GIS, ArcScence™ could be used for 3-D visualization, modeling and analysis of spatially distributed PDB data[71].

Much like bioinformatics, geoinformatics takes the swift processing power and algorithms of computer science to organize information effectively. Hence, the goal is not only just to store data, but to gain knowledge from biological research with special reference to visceral leishmaniasis. However, data in bionformatics are of spatial nature and could be well understood if symbolized, scrutinized and figured out as such geospatial data. In bioinformatics study, geoinformatic can be interactively used for better dynamism, versatility and efficiency. This assists in managing the genome data interactively with the application of superior GIS functionality.

### Bioinformatics and drug discovery: a hope for controlling kala-azar

Bioinformatics is the key to rational drug design. Most drug targets are proteins, so it is important to identify their 3-D structure in detail. Genome projects generate sequence information at a much higher rate than NMR and X-ray laboratories[72]. Small fraction of known X-ray or NMR structures enhances homology modeling to predict 3-D structure based on its template by using bionformatics software. MODELLER (http://www.salilab.org/modeller/) and SWISS-MODEL (http://swissmodel.expasy.org/) are some well-known tools. A number of modeled structures of proteins of *Leishmania* have shown a better choice for obtaining three-dimensional coordinates for proteins[73]. Bionformatics based virtual screening analysis has shown that sulphoraphane, an anticancer compound, has been implicated in the treatment of kala-azar and 3-D model of SIR2 in *L. major* have shown several potentially important structural differences in the nicotinamide binding catalytic domain[74]. These reflective applications and findings of bioinformatics has led to a cost and time effective research. Software like Insight II (now incorporated in discovery studio (Accelrys)) and high power computing workstations reduced the number of trials in the screening of drug compounds and helpful to identify potential drug targets for a specific disease. This initiative aims to identify the functions of different proteins and predict their structures so that these could be used as potential targets for developing drugs against *Leishmania*.

Computer-aided drug design (CADD) is another exciting and diverse discipline where various aspects of applied and basic research combine and stimulate each other to discover, enhance and study drugs and related biologically active molecules[75]. CADD methods are heavily dependent on bioinformatics tools, applications and databases. It is highly important to find out the exact compound that strongly binds to the target. Virtual high-throughput screening (vHTS) is one of the methods to search the molecules[76]. All protein targets can be screened against databases of small-molecule compounds. If there is any hit, the particular compound can be extracted for further testing. Virtual high-throughput screening is expected to increase the impact of virtual screening in the drug discovery process[77]. Another challenge is to find promising leads. After the appropriate lead compound is obtained, it is essential to optimize the structure and properties of the potential drug. Usually, optimization includes a number of modifications of the associated compound that explore the lead candidate. Lead optimization tools such as WABE offer a rational approach to drug design (http://demo.eyesopen.com/about/news/press_releases/2004/Wabe13.html). Swiss-PDB is another excellent tool that can predict key physicochemical properties, such as hydrophobicity and polarity that have a profound influence on how drugs bind to proteins (http://spdbv.vital-it.ch/). For avoiding failure of drug candidate numerous bioinformatics softwares are available such as Insight II, Cerius and Discovery studio, which can predict the toxicity (ADMET) and bioactivity[78]. The predictive power of bionformatics software helps to choose only the most promising drug candidates against *Leishmania*.

### Role of bioinformatics to put forward functional genomics in *Leishmania*

Success in decoding the genetic blueprint has led to the post-genomics era, where there is a need for a fusion of information technology with biomedicine. The post-genomic challenge, mostly in the case of human pathogens, is to decode new information in relation to genes, their control pathways, proteins, and their interactions into improved healthcare. Development of several bioinformatics approaches and methodologies leads to the discovery of protein-coding genes[79]-[80]. Algorithms to find out protein-coding genes are mainly based on similarity (extrinsic methods) and algorithm (intrinsic methods)[81]. Each algorithm is designed mainly to detect true positives and to exclude false positives.

Modern machine learning methodologies focus on solving computational problems in molecular biology. Support Vector Machine (SVM), Genetic Algorithm (GA), Artificial Neural Network (ANN) and Hidden Markov Model (HMM) are machine learning approaches that are used for classification and regression analysis of high throughput biological data such as gene expression data[82]. Machine learning approaches are widely used for gene finding, motifs detection and sequence evolution, statistical genetics, genetic polymorphisms (genotypes), single-nucleotide polymorphisms (SNPs), comparative genomie hybridization (CGH) and their analysis. Consensus epitope predictions from more than 8,271 annotated protein sequences of *L. major* with 5-8 different algorithms including some machine learning algorithms allowed the identification of 78 class I CD8^+^ epitopes and have opened opportunities for the identification of targets for vaccine development[83]. Hidden Markov models and the Viterbi algorithm are also applied to integrated bioinformatics analyses of putative flagellar actin-interacting proteins in *Leishmania* species[84]. These are some bright examples of how machine learning approach and bionformatics analysis move forward the functional genomics of *Leishmania* species.

Bioinformatics tools and techniques learned are now being applied to comparative genomics of *Leishmania*. Finding out the reason of such diverse diseases by different *Leishmania* strains have been an elusive and striking goal for many parasitologists. Comparative genomics by using bioinformatics tools and techniques may provide new hints to understand the disease causing mechanisms of a range of species.

### Future of RS and GBS

The speed of computation is rising due to the development of the tools and techniques of bionformatics and geoinformatics for the management and analysis of biological data. The combination of several bioinformatics databases, software and visualization techniques of GIS may be helpful to identify biological trends and relationships. The integration of various data sources such as clinical, epidemiological, genomics and proteomics data will allow kala-azar specialists to use disease symptoms related to kala-azar to predict and choose better future medicines. GBS can boost the discovery rate in bioinformatics, in large-scale comparative genomics. Intelligent GBS techniques can compute fast, exact and error-free results when utilized in existing bioinformatics system like monitoring, quantifying and predicting the impact of environment phenomena (type of weather changes, and emerging infectious diseases) on human-health. In public health, GIS can play a vital role to resolve issues that required spatial analysis and spatial attention. Hence, kala-azar specialists along with epidemiologists will now be able to predict disease outbreaks by looking at the scenario of spatial data (GIS data) and bioinformatics data.

## CONCLUSION

Presently, both GIS and bioinformatics tools should try to hybridize and develop a programe for better representation of the genome at a higher level of organization, i.e. chromosome and cellular DNA. GIS tries to look at the DNA at the macro level whereas the bioinformatics softwares try to look at the genome at the micro level. Future clinicians targeting for better medicine for any particular disease may be able to look at the changes of the genome of pathogen by collaborative geo-bioinfo-softwares. Hence, future clinician may be able to prescribe appropriate drug to a patient rather than putting the patient in adverse situation which is caused by presently used medicines. Algorithms used for geographical information analysis by GIS software may be implicated in analysis of biological information; hence, specific tools might have to be designed for it.

## References

[1] Desjeux P (1996). Leishmaniasis: Public health aspects and control. Clin Dermatol.

[2] Ministry of Health and Family Welfare. Government of India, 1999. http://www.mohfw.nic.in/.

[3] Leishman WB (1903). On the possibility of the occurrence of trypanosomiasis in India. Br Med J.

[4] Sen Gupta PC (1947). History of kala-azar in India. Indian Med Gaz.

[5] Dutta M, Ghosh TK, Mahajan RC (1983). Review of current status of leishmaniasis epidemiology. Indian Council of Medical Research.

[6] Bora D (1999). Epidemiology of visceral leishmaniasis in India. Nat Med J India.

[7] Alvar J, Yactayo S, Bern C (2006). Leishmaniasis and poverty. Trends Parasitol.

[8] Bhunia GS, Kesari S, Jeyaram A, Kumar V, Das P (2010). Influence of topography on the endemicity of Kala-azar: a study based on remote sensing and geographical information system. Geospatial Health.

[9] Herbretreau V, Salem G, Souris M, Huggot JP, Gonzalez JP (2007). Thirty years of use and improvement of remote sensing, applied to epidemiology: From early promises to lasting frustration. Health Place.

[10] Kalluri S, Gilruth P, Rogers D, Szczur M (2007). Surveillance of arthropod vector-borne infectious diseases using remote sensing techniques: a review. PLoS Pathog.

[11] Curran PJ, Atkinson PM, Foody GM, Milton EJ (2000). Linking remote sensing, land cover and disease. Adv Parasitol.

[12] Hay SI, Packer MJ, Rogers DJ (1997). The impact on remote sensing on the study and control of invertebrate intermediate hosts and vectors for disease. Int J Remote Sens.

[13] Neto JC, Werneck GL, Nery Costa CH (2009). Factors associated with the incidence of urban visceral leishmaniasis: an ecological study in Teresina, Piauí State, Brazil. Cad Saude Publica.

[14] Cross ER, Newcomb WW, Tucker CJ (1996). Use of weather data and remote sensing to predict the geographic and seasonal distribution of Phlebotomus papatasi in southwest Asia. Am J Trop Med Hyg.

[15] Swaminath CS, Short HE, Anderson LAP (1942). Transmission of Indian kala-azar to man by the bite of P. argentipes. Indian J Med Res.

[16] Sharma U, Singh S (2008). Insect vectors of Leishmania: distribution, physiology and their control. J Vector Borne Dis.

[17] Sudhakar S, Srinivas T, Palit A, Kar SK, Battacharya SK (2006). Mapping of risk prone areas of kala-azar (Visceral leishmaniasis) in parts of Bihar state, India: an RS and GIS approach. J Vect Borne Dis.

[18] Gebre-Michael T, Malone JB, Balkewa M, Alia A, Berhe N, Hailu A (2004). Mapping the potential distribution of Phlebotomus martini and P. orientalis (Diptera: Psychodidae), vectors of kala-azar in East Africa by use of geographic information systems. Acta Tropica.

[19] Maguire JH, Costa CH, Lamoniere D (1996). Application of remote sensing and geographical information systems (GIS). A new technology to study the transmission of Leishmania Chagasi in Teresina, Piauí, Brasil. In: An. VL Symp Rem Sens Pat Infec Dis.

[20] Carneiro DDMT, Bavia ME, Rocha W, Lobāo J, Madureira C, Oliveira JB (2004). Identificaçā deáreas de risco para a leishmaniose visceral Americana, através de estudos epidemiológicos e sensoriamento remote orbital em feira de Santana, Bahia, Brasil (2000-2002). Rev Bai Sau Pub.

[21] Clarke KC, McLafferty SL, Tempalski BJ (1996). On epidemiology and geographic information systems: a review and discussion of future directions. Emerg Infect Dis.

[22] Kolaczinski JH, Hope A, Ruiz JA, Rumunu J, Richer M, Seaman J (2008). Kala-azar Epidemiology and Control, Southern Sudan. Emerg Infect Dis.

[23] Alvar J, Bashaye S, Argaw D, Cruz I, Aparicio Pilar, Kassa A (2007). Kala-Azar Outbreak in Libo Kemkem, Ethiopia: Epidemiologic and Parasitologic Assessment. Am J Trop Med Hyg.

[24] Margonari C, Freitas CR, Ribeiro RC, Moura ACM, Timbó M, Gripp AH (2006). Epidemiology of visceral leishmaniasis through spatial analysis, in Belo Horizonte municipality, state of Minas Gerais, Brazil. Mem. Inst. Oswaldo Cruz. Rio de Janeiro.

[25] Elnaiem DA, Schorscher J, Bendall A, Obsomer V, Osman ME, Mekkawi AM (2003). Risk mapping of visceral leishmaniasis: the role of local variation in rainfall and altitude on the presence and incidence of kala-azar in eastern sudan. Am J Trop Med Hyg.

[26] Cardenas R, Sandoval CM, Rodríguez-Morales AJ, Franco-Paredes C (2006). Impact of climate variability in the occurrence of leishmaniasis in Northeastern Colombia. Am J Trop Med Hyg.

[27] Salomon OD, Orellano PW, Lamfrfi M, Scavuzzo M, dri L, Farace MI (2006). Phlebotomine spatial distribution associated with a focus of tegumentary leishmaniasis in Las Lomitas, Formosa, Argentina, 2002. Mem Inst Oswaldo Cruz, Rio de Janerio.

[28] Desjeux P (2001). The increase in risk factors for leishmaniasis worldwide. Trans R Soc Trop Med Hyg.

[29] Clements ACA, Lwambo NJS, Blair L, Nyandindi U, Kaatano G, Kinung'hi S (2006). Bayesian spatial analysis and disease mapping: tools to enhance planning and implementation of a schistosomiasis control programme in Tanzania. Trop Med Int Health.

[30] Hailu A, Mudawi MA, Royce C, Wasunna M (2005). Visceral Leishmaniasis: New Health Tools Are Needed. PLoS Med.

[31] Ali M, Horikoshi Y (2002). Situation analysis of health management information system in Pakistan. Pakistan J Med Res.

[32] Dash A, Srivastava A, Nagpal BN, Saxena R, Gupta SK (2009). Geographical information system (GIS) in decision support to control malaria – a case study of Koraput district in Orissa, India. J Vector Borne Dis.

[33] Srivastava A, Nagpal BN, Joshi PL, Paliwal JC, Dash AP (2009). Identification of malaria hot spots for focused intervention in tribal state of India: a GIS based approach. Int J Health Geogr.

[34] Sabesan S, Raju KHK (2005). GIS for rural health and sustainable development in India, with special reference to vector-borne diseases. Curr Sci.

[35] Raban MZ, Dandona R, Dandona L (2009). Essential health information available for India in the public domain on the internet. BMC Public Healt.

[36] Moss MP, Schell MC, Goins RT (2006). Using GIS in a First National Mapping of Functional Disability among Older American Indians and Alaska Natives from the 2000 Census. Int J Health Geogr.

[37] Yadav SK (2004). Health for All in New Millennium – Is This Possible Without GIS Applications?. J Hum Ecol.

[38] English D, Elliot P, Cuzick J, English D, Stern R (1992). Geographical epidemiology and ecological studies. Geographical and Environmental Epidemiology: Methods for Small-Area Studies.

[39] Werneck GL, Maguire JH (2002). Modelagem especial utilizando modelos mistos: um estudo ecológico sobre Leishmaniose Visceral em Teresina, Piauí, Brasil. Cad Saúde Pública.

[40] Lysenko AJ (1971). Distribution of Leishmaniasis in the old world. Bull. WHO.

[41] Yang GJ, Vounatsou P, Zhou XN, Utzinger J, Tanner M (2005). A review of geographic information system and remote sensing with applications to the epidemiology and control of schistosomiasis in China. Acta Trop.

[42] Raina S, Mahesh DM, Kaul R, Satinder KS, Gupta D (2009). A new focus of visceral leishmaniasis in the Himalayas, India. J Vector Borne Dis.

[43] Thomson MC, Elnaiem DA, Ashford RW, Connor SJ (1999). Towards a Kala-azar risk map for Sudan: mapping the potential distribution of Phlebotomus orientalis using digital data of environmental variables. Trop Med Int Health.

[44] Hati AK (1983). Current status of leishmaniasis—vector biology. Proceedings of the Indo-UK Workshop on Leishmaniasis.

[45] Napier LE (1926). An epidemiological consideration of the transmission of Kala-azar in India. India Med Res Memoir.

[46] Cakan H, Saribas S, Oz V, Polat E, Aslan M, Kocazeybek B (2010). Patients with suspected visceral leishmaniasis in Istanbul. Afr J Micro Res.

[47] Singh SP, Reddy DCS, Mishra RN, Sundar S (2006). Knowledge, attitude, and practices related to kala-azar in a rural area of Bihar state, India. Am J Trop Med Hyg.

[48] Mocan H, Gedik Y, Okten A, Erduran E, Gacar N (1993). Kala-azar in Trabzon (Eastern black sea) region of Turkey. Indian J Pediatr.

[49] Franke CR, Ziller M, Staubach C, Latif M (2002). Impact of El Niño/Southern Oscillation on Visceral Leishmaniasis, Brazil. Emerg Infect Dis.

[50] Ready (2008). Leishmaniasis emergence and climate change. Rev Sci Tech Off Int Epiz PD.

[51] Rajapaksa US, Ihalamulla RL, Udagedera C, Karunaweera ND (2007). Cutaneous leishmaniasis in southern Sri Lanka. Trans R Soc Trop Med Hyg.

[52] Kishore K, Kumar V, Kesari S, Dinesh DS, Kumar AJ, Das P (2006). Vector control in leishmaniasis. Indian J Med Res.

[53] Perry JN, Liebhold AM, Rosenberg MS, Dungan J, Miriti M, Jakomulska A (2002). Illustrations and guidelines for selecting statistical methods for quantifying spatial pattern in ecological data. Ecography.

[54] Wilkinson P, Grundy C, Landon M, Stevenson S, Gatrell AC, Löytönen M (1998). GIS in public health. GIS and Health.

[55] Sipe NG, Dale P (2003). Challenges in using geographic information systems (GIS) to understand and control malaria in Indonesia. Malar J.

[56] Jeanne I (2000). Malaria and schistosomiasis: two examples using systmes of geographical information and teledetection in Madagascar. Bull Soc Path Exot.

[57] Thompson RA, Lima JWO, Maguire JH, Braud DH, School DT (2002). Climatic and demographic determinants of American visceral leishmaniasis in northeastern Brazil using remote sensing technology for environmental characterization of rain and region influences on leishmaniasis. Am J Trop Med Hyg.

[58] Hay S, Snow R, Rogers D (1998). Predicting malaria seasons in Kenya using multitemporal meteorological satellite sensor data. Trans R Soc Trop Med Hyg.

[59] Werneck GL, Costa CHN, Walker AM, David JR, Wand M, Maguire JH (2007). Multilevel modeling of the incidence of visceral leishmaniasis in Teresina, Brazil. Epidemiol Infect.

[60] Fristensky B (2007). BIRCH: A user-oriented, locally-customizable, bioinformatics system. BMC Bioinformatics.

[61] Yu U, Lee SH, Kim YJ, Kim S (2004). Bioinformatics in the Post-genome Era. J Biochem Mol Biol.

[62] Queiroz JW, Dias GH, Nobre ML, Dias MCDS, Araújo SF, Barbosa JD (2010). Geographic Information Systems and Applied Spatial Statistics Are Efficient Tools to Study Hansen's Disease (Leprosy) and to Determine Areas of Greater Risk of Disease. Am J Trop Med Hyg.

[63] Kuonen D (2003). Challenges in bioinformatics for statistical data miners. B Swiss Stat Soc.

[64] Soomro TR, Al-Qaimari G, Wahba H (2008). Geographical Bionformatics Systems. Commun IBIMA.

[65] Pushker R, D'Auria G, Alba-Casado JC, Rodríguez-Valera F (2005). Micro-Mar: a database for dynamic representation of marine microbial biodiversity. BMC Bionformatics.

[66] Ahmad T, Shah AA (2010). remote sensing data dissemination and management: potential of replication and provenance techniques. J Am Sci.

[67] Grady RK (1988). Data Lineage in Land and Geographic Information Systems. Proceedings of GIS/LIS '88, Falls Church, VA:American Congress on Surveying and Mapping.

[68] Bracken I, Webster C (1989). Toward a typology of geographical information systems. Int J Geogr Inform Syst.

[69] Dolan ME, Holden CC, Beard MK, Bult CJ (2006). Genomes as geography: using GIS technology to build interactive genome feature maps. BMC Bioinformatics.

[70] Schweizer P IPK Gatersleben. www.ipk-gatersleben.de/en/02/04/04/.

[71] BSCS (2003). Bioinformatics and the human genome project. A curriculam supplement for high school biology. Developed by BSCS under a contract from the department of Energy. available at: http://www.scribd.com/doc/23653544/tics-and-Human-Genome-Project.

[72] Rodriguez R, Chinea G, Lopez N, Pons T, Vriend G (1998). Homology modeling, model and software evaluation: three related resources. Bioinformatics.

[73] Sahoo GC, Dikhit MR, Rani M, Das P (2009). Homology Modeling and Functional Analysis of LPG2 Protein of Leishmania Strains. J Proteomics Bioinform.

[74] Kadam RU, Kiran VM, Roy N (2006). Comparative protein modeling and surface analysis of Leishmania sirtuin: A potential target for antileishmanial drug discovery. Bioorg Med Chem Lett.

[75] Song CM, Lim SJ, Tong JC (2009). Recent advances in computer-aided drug design. Brief Bioinform.

[76] Subramaniam S, Mehrotra M, Gupta D (2008). Virtual high throughput screening (vHTS) - A perspective. Bioinformation.

[77] Seifert MHJ, Wolf K, Vitt D (2003). Virtual high-throughput in silico Screening. Biosilico.

[78] Shaikh SA, Jain T, Sandhu G, Latha N, Jayaram B (2007). From drug target to Leads-sketching A Physicochemical Pathway for Lead Molecule Design In Silico. Curr Pharm Des.

[79] Burge C, Karlin S (1997). Prediction of complete gene structures in human genomic DNA. J Mol Biol.

[80] Worthey EA, Myler PJ (2005). Protozoan genomes: gene identification and annotation. Int J Parasitol.

[81] Stein LD (2001). Using Perl to facilitate biological analysis. Methods Biochem Anal.

[82] Kasabov N, Sidorov LA, Dimitrov DS (2005). Computational intelligence, bionformatics and computational biology: A brief overview of methods, problems and erspectives. J Comput Theor Nanosci.

[83] Herrera-Najera C, Pine-Aguilar R, Xacur-Garcia F, Ramirez-Sierra MJ, Dumonteil E (2009). Mining the Leishmania genome for novel antigens and vaccine candidates. Proteomics.

[84] Pacheco ACL, Araujo FF, Kamimura MT, Silva SC, Diniz MC, Oliveira FDCE (2009). Hidden Markov models and the Viterbi algorithm applied to integrated bionformatics analyses of putative flagellar actin-interacting proteins in Leishmania spp. Int J Comput Aided Eng Tech.

[85] Nieto P, Malone JB, Bavia ME (2006). Ecological niche modeling for visceral leishmaniasis in the state of Bahia, Brazil, using genetic algorithm for rule-set prediction and growing degree day-water budget analysis. Geospatial Health.

[86] Colacicco-Mayhugh MG, Masuoka PM, Grieco JP (2010). Ecological niche model of Phlebotomus alexandri and P. papatasi (Diptera: Psychodidae) in the Middle East. Int J Health Geogr.

[87] Aparício C, Dantas-Bittencourt M (2003). Análise especial da leishmaniose tegumentar americana. Anais do XI Simpósio Brasileiro de Sensoriamento Remoto Minas Gerais, Brasil.

[88] Fernándeza MS, Salomóna OD, Cavia R, Perez AA, Acardic SA, Guccione JD (2010). Lutzomyia longipalpis spatial distribution and association with environmental variables in an urban focus of visceral leishmaniasis, Misiones, Argentina. Acta Tropica.

[89] Bhunia GS, Kumar V, Kumar AJ, Das P, Kesari S (2010). The use of remote sensing in the identification of the ecoenvironmental factors associated with the risk of human visceral leishmaniasis (kala-azar) on the Gangetic plain, in north-eastern India. Ann Trop Med Parasitol.

[90] Bavia ME, Carneiro DD, Gurgel HC, Madureira-Filho C, Barbosa MG (2005). Remote sensing and geographical information systems and risk of American visceral leishmaniasis in Bahia, Brazil. Parassitologia.

[91] Salomón OD, Quintana MG, Gisela B, Morán ML, Eduardo B, Valdéz DV (2010). Lutzomyia migonei as putative vector of visceral leishmaniasis in La Banda, Argentina. Acta Tropica.

[92] Thompson JD, Higgins DG, Gibson TJ (1994). CLUSTAL W: improving the sensitivity of progressive multiple sequence alignment through sequence weighting, position-specific gap penalties and weight matrix choice. Nucleic Acids Res.

